# Stomatognathic System Changes in Obese Patients Undergoing Bariatric Surgery: A Systematic Review

**DOI:** 10.3390/jpm12101541

**Published:** 2022-09-20

**Authors:** Gerson Fabián Gualdrón-Bobadilla, Anggie Paola Briceño-Martínez, Víctor Caicedo-Téllez, Ginna Pérez-Reyes, Carlos Silva-Paredes, Rina Ortiz-Benavides, Mary Carlota Bernal, Diego Rivera-Porras, Valmore Bermúdez

**Affiliations:** 1 Facultad de Salud, Universidad de Pamplona, Pamplona 543050, Colombia; 2 Facultad de Medicina, Universidad del Zulia, Maracaibo 4002, Venezuela; 3 Facultad de Medicine, Universidad Católica de Cuenca, Cuenca 010109, Ecuador; 4 Facultad de Ingenierías, Universidad Simón Bolívar, Cúcuta 540001, Colombia; 5 Facultad de Ciencias Jurídicas y Sociales, Universidad Simón Bolívar, Cúcuta 540001, Colombia; 6 Facultad de Ciencias de la Salud, Universidad Simón Bolívar, Barranquilla 080022, Colombia

**Keywords:** obesity, bariatric surgery, stomatognathic system, physiology

## Abstract

Background: Obesity is a multifactorial chronic disease involving multiple organs, devices, and systems involving important changes in the stomatognathic system, such as in the orofacial muscles, temporomandibular joint, cheeks, nose, jaw, maxilla, oral cavity, lips, teeth, tongue, hard/soft palate, larynx, and pharynx. Patients with obesity indicated for bariatric surgery reportedly presented with abnormalities in the structures and function of the stomatognathic apparatus. This occurs through the accumulation of adipose tissue in the oral cavity and pharyngeal and laryngeal regions. Therefore, this systematic review aimed to elucidate the changes occurring in the stomatognathic system of patients with obesity after undergoing bariatric surgery. Method: Information was searched based on the equations developed with the descriptors obtained in DECS and MESH using the PRISMA methodology. Studies published between 2010 and October 2021 in databases including PubMed, ProQuest, Scielo, Dialnet, EBSCO, and Springer Link were considered. Results: Eighty articles met the inclusion criteria after evaluating the articles, thereby allowing for the determination of the morphophysiological correlation of the stomatognathic system with the population studied. At the morphological or structural level, changes were observed in the face, nose, cheeks, maxilla, jaw, lips, oral cavity, teeth, tongue, palate, temporomandibular joint, neck, muscles, head, shoulders, larynx, and pharynx. At the morphological level, the main changes occurred in, and the most information was obtained from, the labial structures, teeth, muscles, pharynx, and larynx. Physiological changes were in breathing, phonation, chewing, and swallowing, thereby revealing the imbalance in basic and vital functions. Conclusions: Analyzing the changes and structures of obese patients and candidates for bariatric surgery revealed that, in the preoperative period, the evidence is clear owing to the presence of a wide range of information. However, the information is more limited regarding the postoperative period; thus, further research focusing on characterization of the system postoperatively is warranted.

## 1. Introduction

The stomatognathic system is an integrated and coordinated morpho-functional unit comprising skeletal, muscular, angiological, nervous, glandular, and dental structures organized around the occipito-atloidal, atlantoaxial, cervical vertebral, temporomandibular, dento-dental in occlusion, and dentoalveolar joints, which are organically ligated and functionally related to the digestive, respiratory, phonological, and facial aesthetic expression systems. Consequently, this system is associated with the senses of taste, touch, balance, and orientation. It intervenes in functions of suction, oral digestion (mastication, salivation, tasting, and degradation of carbohydrates), swallowing, verbal communication (phonological modulation, articulation of sounds, speech, and whistles), oral sexuality (smiling, laughing, orofacial gesticulation, and kissing, among other aesthetic-affective manifestations), alternate breathing, and vital defense (coughing, expectorating, sneezing, yawning, sighing, and exhalation and vomiting), which are considered essential for an individual’s survival [1].

Obesity has become a 21st century problem, as well as one of the fastest growing health problems worldwide [2,3]. Currently, it is one of the most important and concerning public health conditions, which is why it has become a priority [4]. According to the Center for Disease Control and Prevention, obesity is defined as “weight above what is adequate or considered healthy given the height of each subject”. Concurrently, it defines it as a chronic disease requiring timely medical care, thereby limiting the activities of daily living. Its main characteristic is excessive accumulation of body fat that has harmful effects on health. However, it is considered a treatable disease [3,5,6].

According to statistics from the World Health Organization (WHO), in 2005, 1.6 billion people aged > 15 years were classified as overweight and 400 million were classified as obese [7]. Currently, there are approximately 1200 million people in the world with problems related to overweight and obesity [8]. This constitutes evidence of high levels of prevalence of the disease, which affects approximately 23% of the adult population of Latin America and the Caribbean, or ~140 million people [9].

However, the Colombian Ministry of Health and Social Protection (MinSalud) estimates that the prevalence of overweight people in Colombia is approximately 56.4% and, thus, it is considered a public health problem for the country. Based on data provided by the same entity, it is predicted that, by the year 2030, 1 out of 2 adults will be obese, and 1 in 4 adults will have severe obesity [10,11]. However, obesity is a preventable disease, and it can be addressed through multidisciplinary intervention, which includes allowing the interaction of several professionals during treatment, involving diet, physical exercise, and pharmacological treatment [12]. However, the combination of eating, sports, and medicinal habits is sometimes not effective or successful. Therefore, patients resort to extreme alternatives, such as bariatric surgery, since it provides an effective solution to the problem in order to reduce food intake and nutrient absorption. This is despite the fact that the procedure includes invasive surgical intervention in the digestive system [2,13]. It is necessary to clarify that not everyone is an ideal candidate for the procedure, even though bariatric surgery is a reliable method for long-term weight loss. Candidates must at least be adults, with a body mass index (BMI) ≥ 40 kg/m^2^ or with a BMI between 35 and 39.9 kg/m^2^ and a severe associated comorbidity [12,13].

Historically, bariatric surgery emerged in the United States in the 1950s. It was the pioneering country in the American continent, followed by Brazil, the second country in the world to perform more bariatric surgeries, with approximately 80,000 surgeries per year [4,14]. For this approach, it must be highlighted that a suitable bariatric technique must primarily be very safe; i.e., with a morbidity of <10% and a mortality of <1%. Second, such a technique must be able to cause the loss of at least 50% of the additional weight, which must be maintained for a period of approximately 5 years, thereby improving the patient’s quality of life. It should be noted that there are three techniques for performing these interventions (malabsorptive, restrictive, and mixed) [15]. The restrictive technique consists of reducing the capacity of the stomach and preventing the passage of food. However, over time, this technique forces patients to undergo a reintervention. The malabsorptive technique reduces the capacity of the stomach by half, producing crossover with the intestine, so that there is a malabsorption of nutrients from food, forcing the patient into restrictive control after surgery. The mixed technique has a restrictive and somewhat malabsorptive character, which allows it to be a well-tolerated procedure, without the patient presenting long-term complications or requiring reintervention [16]. Another technique used to address this problem is sleeve gastrectomy (SG) (the malabsorptive type), and it has been the most commonly performed technique in the world, and in Colombia, since 2004, followed by the Roux-en-Y gastric bypass (RYGB) (the mixed type) [17].

In conclusion, it is important to highlight that, despite the many benefits of making use of this procedure, there are also various changes and anomalies in the structures and functioning of the different systems, including the stomatognathic system [18,19]. The literature and the investigated studies make it clear that these anomalies severely compromise the entire stomatognathic system due to the accumulation of adipose tissue in the oral cavity and in the pharyngeal and laryngeal regions [19], with the adiposity of these regions being the etiology responsible for the changes in these large and important structures of the patient, thus compromising morphology and physiology [20]. Considering the arguments and findings previously revealed, the following research question arises: how are the stomatognathic systems of obese patients, and those of obese patients with bariatric surgery, characterized?

## 2. Materials and Methods

This review was performed following the parameters proposed by the PRISMA methodology. For this, the databases were identified, and thesauruses were defined in the search for information. The studies were also selected based on inclusion and exclusion criteria that facilitated the assessment of the studies’ quality and reliability and that, eventually, allowed answering the research question posed [21,22,23].

The PICO tool was used to construct the research question. This tool was employed owing to the fact that it is used to improve the specificity and conceptual clarity of the clinical problems to be studied, as well as to perform searches with greater quality and precision, which allows for the collection of pertinent and accurate data to answer the problem question [24,25].

### 2.1. Research Question

In accordance with the theme established for the research, the components of the PICO strategy shown in Table 1 were established, resulting in the following research question: how are the stomatognathic systems of obese patients, and those of obese patients with bariatric surgery, characterized?

#### 2.1.1. Inclusion Criteria

1. Overweight subjects, those with obesity or morbid obesity, or those who had undergone bariatric surgery
2. A publication time window of 10 years;
3. Articles focused on the evaluation of aspects related to the distal airways, upper airways, lower airways, stomatognathic system (morphology and physiology), respiratory system, masticatory system, and swallowing mechanics;
4. Studies conducted with humans;
5. Full-text articles;
6. Free-access articles and current DOIs.

#### 2.1.2. Exclusion Criteria

1. Articles with DOIs that were not current within the databases for download;
2. Research with a time window of >10 years;
3. Articles that were not related to human beings;
4. Grey literature, such as theses, white books, research and project reports, annual or activity reports, conference proceedings, preprints, working papers, newsletters, technical reports, recommendations and technical standards, patents, technical notes, data and statistics, presentations, field notes, laboratory research books, abstracts, academic courseware, lecture notes, and evaluations, were excluded.

### 2.2. Sources of Information

The key terms were selected from the Descriptors in Health Sciences (DECS) and the Medical Subject Headings (MESH) (see Table 2).

#### 2.2.1. Search Strategies

A search strategy was developed with the aid of trained institutional professional librarians from la Universidad Simón Bolívar, Colombia, and la Universidad de Pamplona, Colombia.

Subsequently, the search equations were designed with the terms found. These equations were created using the logical operators AND/OR/NOT and symbols such as “” and (). The information search was conducted in PubMed, ProQuest, Scielo, Dialnet, EBSCO, and Springer Link in the English language (see Table 3).

#### 2.2.2. Characteristics of the Studies

Initially, the interventions and the respective descriptions of the treatment therapy were classified. Likewise, these interventions were compared from the perspective of the control and experimental groups—based on the characteristics of the therapies—including the model, the technique (if applicable), whether they involved group or individual interventions, the characteristics of the sessions (number of sessions and frequency and duration of each session), the effectiveness and benefit of therapies, the intervention protocol, randomization, and the characteristics of the participants.

Additionally, the characteristics of the therapists and evaluators of the results, the follow-up in time after the interventions, and the findings of the studies were identified. In cases of missing or unclear data, emails were sent requesting the additional information.

### 2.3. Selection and Analysis

Initially, a preliminary selection of studies based on a review of inclusion criteria, population characteristics, type of study, and year was taken into consideration. Subsequently, a registration table was filled out independently in Excel, prepared by the authors, in which the key elements of each of the selected studies were specified. The process used in the identification, screening, eligibility, and inclusion of articles is briefly described, following the structure proposed by the PRISMA statement [26].

## 3. Results

**Figure 1:**
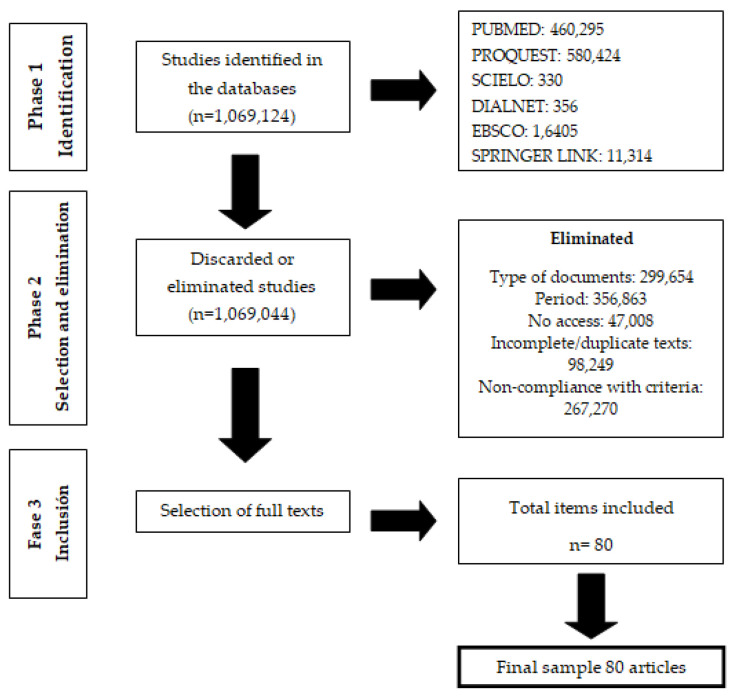
PRISM diagram.

The eligibility criteria were determined following the order established in the methodology by developing each of the phases of the PRISMA flowchart (Figure 1).

### 3.1. Identification Phase

The search was performed in the databases PubMed, ProQuest, Scielo, Dialnet, EBSCO, and Springer Link, according to the crosses of variables constructed from DECS and MESH keywords. Then, the following filters were applied: type of document, time window, full or duplicate text, articles without access, and non-compliance with criteria. Finally, articles were selected to obtain the final sample of 80 articles that were used in this investigation (See Table 4).

### 3.2. Selection and Elimination Phase

The initial selection of the research articles was carried out through the preliminary reading of the titles, summaries, and, later, the introduction, allowing the identification of the most relevant articles regarding the subject under investigation, with a total of 80 selected articles. The results for each variable crossing in English are listed below (see Table 5) for the six PubMed, ProQuest, Scielo, Dialnet, EBSCO, and Springer Link databases.

In the first search, 17 crosses were made in English between the different variables, resulting in 26 articles from PubMed, 24 from ProQuest, 16 from Scielo, 3 from Dialnet, 5 from EBSCO, and 6 from Springer Link, for a total of 80 items.

### 3.3. Inclusion Phase

The selection proceeded after reading the titles and the summaries of the articles, and they were analyzed in their entirety with a complete read-through, applying criteria that allowed a selection that, thus, made it possible to obtain those that clearly answered the question posed initially. The selection corresponded to a final sample of 80 articles (See Table 6).

Table 7 shows the characterization of the stomatognathic system from its morphological changes.

Table 8 presents the characterization of the stomatognathic system from its physiological changes.

## 4. Discussion

The stomatognathic system (SS) is also called the masticatory apparatus (MA), and the word “stomatognathic” originates from the Greek “stoma” (mouth) and “gnathos” (jaw). The stomatognathic system refers to structures that are anatomically and functionally linked [51] to processes related to vital functions, such as breathing, sucking, chewing, and swallowing, and social functions, such as phonation and articulation [78], and these are integrated by different structures that allow the development of each function in a harmonious and balanced way [38,41]. First, there are bony structures, such as the skull, facial bones, hyoid bone, larynx, maxilla, mandible, and bony palate. There are also muscular structures, such as the muscles for mastication and facial expression and the muscles of the tongue, soft palate, pharynx, and neck, as well as other structures, such as the head, nose, oral cavity, teeth, and shoulders [29].

Based on the above, any change or alteration in any of the bodily structures can lead to its imbalance and this will simultaneously have an effect on the performance of its functions, thereby generating a negative influence on people’s daily lives [48]. Currently, studies demonstrate that obesity is the etiology of these structural and physiological changes, given that obese patients present excessive accumulations of adipose tissue in regions that have direct effects [19,75]. To reduce the dysfunctions associated with obesity, these patients undergo surgical interventions, including bariatric surgery, which appears to have a positive impact. However, if a patient does not receive an intervention for existing alterations, the alterations reportedly persist and even worsen [39,50].

Within the investigated databases, it is possible to provide details on the characterization of the SSs of patients with obesity and post-bariatric surgery. In terms of the facial features, patients with obesity may present an asymmetry, with differences in measurements of the middle and lower thirds of the face [18,30], as well as in the corners of the lips in the habitual position and when the subjects smile [70]. A flattened nose, with a possible deviated septum and turbinate hypertrophy caused by the narrowing of the nostrils, may trigger these patients to become oral breathers and affect other SS functions [36,52]. Hypotonic cheeks, with slight sagging on one side and dysfunction in performing requested exercises, such as inflating, retracting, and sucking, may also be present [18]. However, other authors state that the cheek hypertonia of the patients included in their studies was attributable to continuous food intake, an argument contradicted by theory, as it has been determined that obese patients do not perform the chewing phase correctly and have preferences to swallow food whole [30,54].

Atretic maxilla is evidenced when the hard palate is arched or vaulted and the soft palate, for its part, is increased in length and reduced in mobility, as well as being characterized by mobility alterations in the mandible, with rotation of the mandibular angle [34], and repercussions related to the presence of noises from the temporomandibular joint. Lips show the presence of dryness and are contracted during the swallowing process. In the usual state, they do not present a lip seal, their tone is decreased [54,72], and they exhibit dysfunction when performing requested praxias (protruding, retracting, and lateralizing to both sides) [18]. The upper lip, for its part, is short and hypo-functional, and the lower lip is observed to have great volume [30].

Regarding the oral cavity, a study based on the Mallampati scale—which is useful for analyzing air obstructions that prevent its passage from the nose and mouth to the lower respiratory tract—reported that the results obtained by these patients were Class III (indicating visualization of only the soft palate and uvula) and Class IV (indicating visualization only of the hard palate) [20], states that may indicate the appearance of obstructive sleep apnea [34]. Authors emphasize that the results of this scale in the case of a post-bariatric patient will remain unchanged, since such changes only depend on the gradual loss and reduction of the BMI, as structures such as the larynx and pharynx in obese patients are affected through the accumulation of fat in the respiratory tract, thickening and narrowing the lateral walls and obstructing the nasopharyngeal mechanics (adenotonsillar hypertrophy), which is why pharyngeal collapse can occur [20,36,81].

Dental wear is the gradual loss of tooth substance without the involvement of the caries process or interference from the action of microorganisms or trauma. Changes in lifestyle, diet, and behavior play a fundamental role in this process. Patients with obesity usually consume unhealthy diets and, therefore, have an oral profile including loss of teeth [54], erosion, attrition, abrasion, and dental fraction [58], as well as other characteristics independent of their diet, such as dental caries, periodontitis [2,32,66], open bite, and upper teeth protrusion [30]. Patients undergoing RYGB present recurrent acidity in the oral cavity compared to those undergoing SG, affecting dental erosion, periodontal disease, and hypersensitivity [2,58].

The orofacial muscles are crucial for the performance of stomatognathic functions that are relevant to health and quality of life. Therefore, for example, problems may arise in chewing and in the manipulation and propulsion of the food bolus during swallowing if performance is affected [74]. In the case of patients with obesity, their musculatures will be perceived as hypotonic with hyperfunction of the mentalis muscle and tension during swallowing [30], excessive contraction of the orbicularis oris [54], and hypotonicity of the temporalis muscle [70]. Regarding the musculature of the tongue, some studies register an increased tone [52,70]; however, most studies agree that it is hypotonic [30,54,72]. In addition, an increase in volume and an abnormal position, called “cloaked or low tongue”, or sometimes interposed between the dental arches [18,20,54], can be observed.

Finally, structures such as the head, neck, and shoulders are also highlighted within the information obtained from the studies, since functional harmony between them is necessary to ensure that each of the functions involved in the SS is correctly developed. When patients with obesity accumulate fat, the circumference of their neck increases, which is considered an anthropometric predictor of the severity of obstructive sleep apnea syndrome and of a possible collapse of the upper airway [42,52]. In addition, these patients adopt a head tilt and hyperflexion posture, with one shoulder more inclined relative to the other [30].

Undoubtedly, the results obtained reflect a broad characterization of the functions of the SS in obesity. Before bariatric surgery, patients with obesity suffer from respiratory disorders, such as alveolar hypoventilation and obstructive sleep apnea [63], apnea being a condition that has been reported on multiple occasions with a wide variety of evidence in scientific articles [13,18,20,44,57,60,82]. The respiratory mode of obese patients is oral because of the numerous structural changes; a chronic nasal obstruction is possibly maintained, thereby leading to attempts to restore the function that is vital for the patient’s survival and, consequently, affecting physiological breathing [18,36]. Likewise, there is an insufficient ability to perceive odors from the environment [30]. In addition to this, the collapse in the structures that make up the upper airway [42] may lead to respiratory failure and other lung diseases, such as asthma [20]. When there is an obstruction of the flow of air coming from the pulmonary complex, the vibration of the vocal folds and the number of times per second in which they must vibrate are affected, which is known as a decreased fundamental frequency. Phonorespiratory incoordination occurs because of poor air support as a result of affected lung capacity, which leads to alterations in vocal mechanics [52].

Episodes of snoring are frequent and are the result of limitations related to and increases in respiratory effort, causing hypoventilation or slow breathing and generating sleep interruption, also known as apnea [36]. Likewise, patients experience moderate dyspnea or a feeling of shortness of breath [44], which can, in extreme circumstances, trigger hypoxia, decreased oxygen supply to various tissues, or low desaturation [57] and reduced residual lung capacity. This indicates inadequate chest expansion and can, therefore, affect normal respiratory function [76]. Obstructive sleep apnea persists even after bariatric surgery; however, the development of adult respiratory distress syndrome is also possible [61]. In contrast, some bibliographic bases show the existence of a significant reduction in the rate of obstructive sleep apnea and hypopnea by ≥50% and of nocturnal episodes to <20 events per hour, which can be characterized as adequate or within a range of possible normality [57].

In the phonatory field, one study reports, through a speech evaluation, an air leak during the emission of phonemes, with mandibular deviation during spontaneous speech [70], as well as a short maximum phonation time, justified because ventilatory pressures are lower than expected during inspiration and expiration, causing a reduction in respiratory muscle strength, lung capacity, and respiratory reserve volume [11]. Obese patients present with fatigue and phonatory dysfunction because of the dehydration of the mucosa of the area, generating altered vocal qualities (strangled, hoarse, and gasping voice), the presence of murmurs, vocal instability, nervousness, and prosodic alteration of speech or vocal brightness [52]. However, these patients do not report significant improvements in the maximum phonation time after undergoing the surgical procedure [11].

The masticatory patterns of patients with obesity undergoing bariatric surgery are strongly affected. The characteristics are maintained before and after bariatric surgery, which means that the masticatory dysfunction does not change and is persistent. This dysfunction is caused by dental alterations, the cause of which is, theoretically, the high level of recurrent acidity in the oral cavity, which causes caries, erosion, or loss of teeth and dental hypersensitivity [2], as well as, simultaneously, a weak bite force. There is also a chronic unilateral preference [18,73] and mild mucosa in the dehydrated area [40], without a crushing phase, according to the food bolus [18], and hypotonicity, or low muscle tone, in the lips and tongue [28]. Chewing speed is also affected [69,70], and some patients show the ability to taste outside the normal parameters or decreased ability to taste [30] and low production of saliva or hyposalivation, which leads to difficulty in moistening and macerating food adequately and satisfactorily [40].

The swallowing function of patients with obesity, like the other vital functions, is also affected owing to the tension in the facial muscles and the abnormal position of the tongue during chewing (hooded or lowered tongue). The information that suggests that obese patients swallow repeatedly or abnormally is new, with alterations of 50% for swallowing solid consistencies and 25% for swallowing liquid consistencies [18]. The mucosa that converges in the process of swallowing food is dehydrated [4]. The swallowing pattern is classified as having low efficiency because of repeated swallowing of the bolus or multiple swallows [18]. These changes make it difficult to propel the food orally, in addition to the existence of pharyngeal, nasal, or palatal obstructions [30]. Patients may also show food residues in the oral cavity [70], a large food bolus construct [27,31], gastroesophageal reflux disease [32,44,83], and oropharyngeal dysphagia (OFD) [55], a symptom that refers to the difficulty in forming or moving the food bolus toward the pharyngeal wall or toward the esophagus and which is concurrently related to difficulty in oral propulsion. OFD causes alterations in safety (possible aspiration pneumonia) and efficacy (dehydration and malnutrition), thereby increasing the morbidity and mortality of individuals experiencing this condition and, consequently, deteriorating their quality of life [46,73]. Other features of the swallowing function in obese patients include esophagitis or inflammation, which damages the duct that extends from the throat to the stomach [83], and binge eating disorder or compulsive behavior through binge eating, where the main characteristic is loss of control over what is eaten [43,71]. The swallowing reflex is well coordinated with breathing patterns in normal humans. However, patients with obstructive sleep apnea syndrome may have a swallowing disorder that reflects abnormal nerve and muscle function in the suprapharynx [34].

Furthermore, the observation that patients with obesity have a nutcracker-shaped esophagus is frequently related to an anomaly or to hypercontractile motor disorder with characteristically high amplitude waves in the distal esophagus—the main symptoms being chest pain and dysphagia [81]. All the abovementioned alterations are categorized under the terms “swallowing dysfunction” and “adapted swallowing”. Theory defines adapted or atypical swallowing as an alteration in the oral phase of swallowing that is characterized by the inadequate position of the tongue and other structures of the oral cavity and that appears when there is an alteration in the form and function of the same. This altered pattern is observed when the structures of the oral cavity have to adapt as a consequence of a structural or functional alteration [60]. Sensations of choking or stagnation [18], gastroesophageal reflux [2,18], and binge eating disorder [43,71] are alterations that, according to theory, continue to present even after the postoperative period, which indicates that swallowing dysfunction persists after bariatric surgery.

In addition, the reviewed studies show the mechanical effects of obesity on pulmonary physiology and the function of adipose tissue, this latter being an endocrine organ that produces systemic inflammation and affects central respiratory control [49]. Patients with morbid obesity show increased total, airway, peripheral, and tissue system resistance, even though they do not show limitations in expiratory flow or reduced respiratory muscle strength [63]. The already mentioned mechanical effects on the respiratory system can trigger dyspnea, wheezing, and cough, thereby becoming a morbidity of the respiratory system [47]. Major respiratory complications of obesity are considered to include increased ventilatory demand, increased effort in breathing, respiratory muscle inefficiency, and decreased respiratory compliance [48].

Changes in respiratory system compliance and lung volumes can negatively affect pulmonary gas exchange and lead to upper airway obstruction and sleep-disordered breathing. Therefore, the perioperative period should be carefully observed [82]. Among other things, decreased functional residual capacity, decreased expiratory reserve volume, decreased compliance, and increased resistance of the respiratory system imply breathing with low lung volume, promoting airway closure in dependent lung zones with consequent abnormalities in gas exchange, even though the capacity of the lungs to diffuse carbon monoxide is normal or increased [83].

Obesity is characterized by increased systemic and pulmonary blood volume (pulmonary vascular congestion). The concomitant abnormal diffusion of the alveolar membrane suggests subclinical interstitial edema. In this setting, functional abnormalities should encompass the entire distal lung, including the airways [38]. These abnormalities are caused by the reduction in lung volume at rest; however, airway inflammation, vascular congestion, and/or concomitant intrinsic airway disease may occur [41]. A study of obese women demonstrated that the airways are characterized by hyperreactivity. Bronchial hyperreactivity is an exaggerated response of the bronchial mucosa and is the cause of bronchospasm. Some of the agents that can trigger bronchial hyperreactivity are respiratory infections, substances present in the environment, such as pollen or smoke, and certain drugs [72].

Global dysfunction of the distal lung (alveolar membrane and distal airways) is associated with pulmonary vascular congestion and failure to reach the high-output state of obesity. Pulmonary vascular congestion and consequent fluid transudation and/or alterations in alveolar-capillary membrane structure can be considered as often unrecognized causes of airway dysfunction in obesity [41].

One study describes the phenotype of pulmonary dysfunction in obesity as reduced functional residual capacity (FRC) with airway narrowing, distal respiratory dysfunction, and bronchodilator response [38]. The repercussions of obesity on respiratory function are associated, above all, with the restrictive alteration caused by excess adipose tissue. Increased fat in the chest and abdomen can shift the elastic equilibrium point between the chest and lungs, thereby reducing FRC [42]. Obesity also significantly interferes with respiratory function by decreasing lung volume, particularly expiratory reserve volume, and FRC [84].

For this reason, bariatric surgery effectively reduces neck and waist circumference, increases peak ventilatory pressures, improves sleep architecture, and reduces sleep-disordered breathing, specifically obstructive sleep apnea, in patients with severe obesity [80].

## 5. Conclusions

There are compromises that negatively affect the harmonious and joint form of the morphological and physiological unit that is the stomatognathic system (SS) in patients with obesity, which are due to an imbalance caused by the concentrated accumulation of adipose tissue characteristic of a BMI ≥ 40 kg/m^2^ or between 35 and 39.9 kg/m^2^ above that established for normality according to an individual’s height.

Breathing, chewing, and swallowing are the functions of the stomatognathic system in patients with obesity that provided the most information during the evidence search process. Most of the articles investigated coincided in specific information, which allowed the broad characterization of each of these functions and, in the same way, of those structural alterations that play a significant role in said functions. The most cited were lip dysfunction; cloaked tongue, or lingual and cheek hypotonia; lip hypotonia with no lip seal; the presence of periodontitis; dysfunction in the width and height of the hard palate; and, finally, thickening of the lateral walls of the pharynx.

Swallowing dysfunctions caused by gastroesophageal reflux may vary according to the bariatric procedure performed. If the patient undergoes RYGB, acid reflux will decrease, given the implications of the procedure, unlike if they undergo SG, which favors an increase in acid reflux in these patients and, simultaneously, the alteration of the stomatognathic system (SS) at the morphological level.

The information evidenced within the literature reflects the need to develop and implement multidisciplinary work with obese patients before and after they undergo bariatric surgery, given that the alterations of the stomatognathic system, if not treated in the preoperative period, may persist and even worse, thereby negatively impacting these patients’ quality of life.

After performing an exhaustive search on the stomatognathic system in patients with obesity before and after surgery and analyzing all the information obtained on the structural and functional alterations of this system, we noted that there is ample and clear evidence regarding the condition of obese patients who are candidates for bariatric surgery. However, when considering post-bariatric patients in relation to the stomatognathic system, the information changes and shows great limitations. Certainly, in this case, the data obtained were scarce.

It is evident how interest in bariatric surgery has grown in recent years. The number of bariatric surgeons has been progressively increasing, along with the number of patients undergoing these surgeries, which highlights considerable positive impacts on weight loss, metabolic control, and self-esteem, among others. However, many other diseases in obese patients that compromise this population’s quality of life, such as those of the SS, have been forgotten, and there is a lack of knowledge about the advantages of this surgery for the SS.

Considering the abovementioned information, the greatest contribution of this review would be to arouse interest in the evaluation of patients with obesity before and after surgery beyond weight control. A comprehensive and multidisciplinary evaluation should be performed that includes breathing, chewing, and swallowing as basic functions to be analyzed, thereby allowing patients to have a great opportunity to improve their quality of life.

## Figures and Tables

**Table 1:** Research Question.

Component	Description
P: Patient or problem of interest (Population)	Obese patient and post-bariatric surgery
I: Intervention	Assessment of the stomatognathic system
C: Comparison	Stomatognathic system
O: Outcome	Alterations or changes in anatomical and functional structures

**Table 2:** DECS and MESH descriptors.

Source	Keyword	Related Terms
DECS	Distal airways	No records found
MESH	Distal airways	No records found
DECS	Obesity	No records found
MESH	Obesity	Morbid obesity, excess adipose tissue, abnormal weight gain
DECS	Overweight	No records found
MESH	Overweight	Excess weight, increased body fat, increased adipose tissue
DECS	Bariatric surgery	No records found
MESH	Bariatric surgery	Weight reduction, metabolic surgery, bariatric surgical procedure, stomach stapling, gastroenterostomy, gastric bypass, gastroplasty, jejunoileal bypass, lobectomy, lipoabdominoplasty
DECS	Upper respiratory tract	No records found
MESH	Upper respiratory tract	Respiratory system, respiratory tract, upper respiratory tract
DECS	Lower respiratory tract	No records found
MESH	Lower respiratory tract	No records found
DECS	Respiratory system	No records found
MESH	Respiratory system	Airways, respiratory function
DECS	Masticatory system	No records found
MESH	Masticatory system	Stomatognathic system
DECS	Masticatory apparatus	No records found
MESH	Masticatory apparatus	No records found
DECS	Masticatory dynamic	No records found
MESH	Masticatory dynamic	No records found
DECS	Swallowing disorder	No records found
MESH	Swallowing disorder	Swallowing disorder, difficulty swallowing, dysphagia
DECS	Swallowing reflex	No records found
MESH	Swallowing reflex	No records found
DECS	Swallowing physiology	No records found
MESH	Swallowing physiology	No records found
DECS	Swallowing biomechanics	No records found
MESH	Swallowing biomechanics	No records found
DECS	Dysphagia	No records found
MESH	Dysphagia	Swallowing disorder, neuromuscular disorder, or mechanical obstruction
DECS	Aspiration	No records found
MESH	Aspiration	Pneumonia, respiratory aspiration
DECS	Myofunctional disorder	No records found
MESH	Myofunctional disorder	No records found
DECS	Orofacial disorder	No records found
MESH	Orofacial disorder	No records found
DECS	Swallowing	No records found
MESH	Swallowing	Swallow
DECS	Masticatory alteration	No records found
MESH	Masticatory alteration	No records found
DECS	Orofacial motor skills	No records found
MESH	Orofacial motor skills	No records found
DECS	Myofunctional therapy	No records found
MESH	Myofunctional therapy	Orofacial myotherapy, orofacial myologies
DECS	Stomatognathic system	No records found
MESH	Stomatognathic system	No records found
DECS	Breathing	No records found
MESH	Breathing	Breath work
DECS	Suction	No records found
MESH	Suction	No records found
DECS	Speech	No records found
MESH	Speech	Verbal communication
DECS	Phonation	No records found
MESH	Phonation	Sound production
DECS	Chewing	No records found
MESH	Chewing	No records found

Source: Information obtained from DECS and MESH.

**Table 3:** Search equations.

Database	Search Algorithm
PubMed, ProQuest, Scielo, Dialnet, EBSCO, and Springer Link	(“Bariatric Surgery”) AND (“Disorders”) AND (“Myofunctional”)
	(“Bariatric Surgery”) AND (“Disorders”) AND (“Myofunctional” OR “Orofacial”) OR (“Disorder Physiology”) AND (“Obesity”)
	(“Alteration”) AND (“Masticatory System”) AND (“bariatric surgery” OR “Obese”)
	(“Deglutition”) AND (“bariatric surgery” OR “Obese”)
	(“Orofacial Motor Skills”) AND (“Physiology”)
	(“Myofunctional Therapy” OR “Stomatognathic System”) AND (“Physiology”)
	(“Orofacial”) AND (“Disorder”) AND (“bariatric surgery”) AND (“Respiration” OR “Suction” OR “Swallowing” OR “Speech” OR “Phonation”)
	(“Orofacial” OR “Bariatric Surgery”) AND (“Breathing”)
	(“Orofacial”) AND (“Disorder”) AND (“Bariatric Surgery”) AND (“Suction”)
	(“Disorder”) AND (“Bariatric Surgery”) AND (“Swallowing”)
	(“Bariatric Surgery”) AND (“Speech”)
	(“Disorder”) AND (“Bariatric Surgery”) AND (“Phonation”)
	(“Orofacial”) AND (“Disorder”) AND (“Obesity”) AND (“Breathing”)
	(“Orofacial”) AND (“Disorder”) AND (“Obesity”) AND (“Suction”)
	(“Orofacial”) AND (“Obesity”) AND (“Swallowing”)
	(“Orofacial”) AND (“Disorder”) AND (“Obesity”) AND (“Speech”)
	(“Orofacial”) AND (“Disorder”) AND (“Obesity”) AND (“Phonation”)
	(“Distal Airways”) AND (“Obesity”)
	(“Distal Airways”) AND (“Overweight”)
	(“Distal Airways”) AND (“Obesity”) AND (“Bariatric Surgery”)
	(“Distal Airways”) AND (“Overweight”) AND (“Bariatric Surgery”)
	(“Upper Airways”) AND (“Obesity”)
	(“Upper Respiratory Tract”) AND (“Overweight”)
	(“Upper Airway”) AND (“Overweight”) AND (“Bariatric Surgery”)
	(“Lower Respiratory Tract”) AND (“Obesity”)
	(“Lower Respiratory”) AND (“Overweight”)
	(“Lower Respiratory Tract”) AND (“Obesity”) AND (“Bariatric Surgery”)
	(“Respiratory System”) AND (“Obesity”)
	(“Masticatory System”) AND (“Obesity”)
	(“Masticatory Apparatus”) AND (“Obesity”)
	(“Masticatory Dynamics”) AND (“Obesity”)
	(“Swallowing Disorder”) AND (“Obesity”)
	(“Swallowing Reflex”) AND (“Obesity”)
	(“Swallowing Physiology”) AND (“Obesity”)
	(“Swallowing Biomechanics”) AND (“Obesity”)
	(“Dysphagia”) AND (“Obesity”)
	(“Aspiration”) AND (“Obesity”)

**Table 4:** Filters applied.

Database	Total Articles	Type of Document	Period	Incomplete and/or Duplicate Texts	No Access	Non-Compliance with Criteria	Selected Articles
PUBMED	460,295	29,752	330,324	79,016	20,345	832	26
PROQUEST	580,424	259,904	18,580	18,667	24,348	258,901	24
SCIELO	330	120	36	32	15	111	16
DIALNET	356	43	43	1	0	266	3
EBSCO	16,405	5280	4012	3	0	7105	5
SPRINGER LINK	11,314	4555	3868	530	2300	55	6
TOTAL	1,069,124	299,654	356,863	98,249	47,008	267,270	80

**Table 5:** Results of the English language crosses in the databases.

Matches/Databases	PUBMED	PROQUEST	SCIELO	DIALNET	EBSCO	SPRINGER LINK
Obesity + Bariatric Surgery	7	8	3	1	1	1
Obesity + Stomatognathic System	6	6	5	2	1	2
Obesity + Physiology	9	6	8	0	3	3
Obesity + Upper Airway	2	2	0	0	0	0
Obesity + Lower Airway	2	2	0	0	0	0
Total	26	24	16	3	5	6

**Table 6:** Study selection.

N	Database	Title	Author	Year	URL
1	DIALNET	Standardized care plan in bariatric surgery [27]	Mesa García, C; Muñoz Del Castillo, M.	2016	https://dialnet-unirioja-es.unipamplona.basesdedatosezproxy.com/servlet/articulo?codigo=7801587 (accessed on 23 November 2021)
2	DIALNET	Formulation of criteria to record tongue position in patients with atypical swallowing [28]	Pachon Salem, L. E.	2016	https://dialnet.unirioja.es/descarga/articulo/6045809.pdf (accessed on 23 November 2021)
3	DIALNET	Structural and Functional Alterations of the Stomatognathic System: Speech-Language Management [29]	Pérez Serey, J; Hernández Mosqueira, C; Fuenzalida Cabezas, R.	2021	https://arete.ibero.edu.co/article/view/art.17105 (accessed on 23 November 2021)
4	EBSCO	Myofunctional and electromyographic characteristics of obese adolescent children [30]	Bolzan Berlese, D; Copetti, F; Maciel Weimmann, A; Fantinel Ferreira, P; Bonfanti Haeffner, L.	2013	https://web-s-ebscohost-com.ezproxy.uniminuto.edu/ehost/detail/detail?vid=0&sid=97de324b-fcf4-47c4-a413-4b7a6cde6c62%40redis&bdata=Jmxhbmc9ZXMmc2l0ZT1laG9zdC1saXZlJnNjb3BlPXNpdGU%3d#AN=90594980&db=a9h (accessed on 04 December 2021)
5	EBSCO	Pulmonary Function and Obesity [31]	Carpio, C., Santiago, A., García De Lorenzo, A; Álvarez-Sala, R	2014	https://scielo.isciii.es/scielo.php?script=sci_arttext&pid=S0212-16112014001200009 (accessed on 04 December 2021)
6	EBSCO	Characterization of sleep disorders, snoring, and alterations of the stomatognathic system of obese candidates for bariatric surgery [20]	Mores, R; Delgado, S. E; Martins, N. F; Anderle, P; Da Silva Longaray, C; Pasqualeto, V. M; Batista Berbert, M. C.	2017	http://www.rbone.com.br/index.php/rbone/article/view/447 (accessed on 04 December 2021)
7	EBSCO	Maximum phonation time in people with obesity not undergoing or undergoing bariatric surgery [11]	Fonseca, A; Salgado, W; Dantas, R.	2019	https://www.hindawi.com/journals/jobe/2019/5903621/ (accessed on 04 December 2021)
8	EBSCO	Obesity, bariatric surgery, and the impact on oral health: A review of the literature [32]	Mosquim, V; Aparecido Foratori, J. G; Saory Hissano, W; Wang, L; Sales Peres, S.	2019	https://pesquisa.bvsalud.org/portal/resource/pt/biblio-1051047 (accessed on 04 December 2021)
9	PROQUEST	Impaired swallowing reflex in patients with obstructive sleep apnea syndrome [33]	Teramoto, S; Sudo, E; Matsuse, T; Ohga, E.	1999	https://www.proquest.com/docview/200498630/9EDD348CC9A4A96PQ/2?accountid=48797&forcedol=true (accessed on 17 December 2021)
10	PROQUEST	The stomatognathic system and body scheme [1]	Barreto, J. F	1999	https://www.redalyc.org/pdf/283/28330405.pdf (accessed on 17 December 2021)
11	PROQUEST	Obesity and the lungs: 2 · Obesity and sleep-disordered breathing [34]	Crummy, F; Piper, A. J; Naughton, M. T	2008	https://www.proquest.com/docview/1781775154/FFC00B28C8B14725PQ/73?accountid=48797&forcedol=true&forcedol=true (accessed on 17 December 2021)
12	PROQUEST	The effect of dental status on changes in chewing in obese patients after bariatric surgery [35]	Godlewski, A. E; Veyrune, J; Ciangura, C; Chaussain, C.	2011	https://www.proquest.com/docview/1306252768/9EDD348CC9A4A96PQ/4?accountid=48797&forcedol=true (accessed on 17 December 2021)
13	PROQUEST	Habitual snoring and atopic status: Correlations with respiratory function and tooth occlusion [36]	Zicari, A. M; Marzo, G; Rugiano, A; Celani, C; Carbone, M. P.	2012	https://www.proquest.com/docview/1197719038/8A83C372D8DF4F3DPQ/9 (accessed on 17 December 2021)
14	PROQUEST	Impairment of the distal airway in normally reactive obese women [37]	Marin, G; Gamez, A. S; Molinari, N; Kacimi, D; Vachier, I.	2013	https://www.proquest.com/docview/1434621519/2E1BA5ED485B4119PQ/4?accountid=48797 (accessed on 17 December 2021)
15	PROQUEST	Airway dysfunction in obesity: The response to voluntary restoration of end-expiratory lung volume [38]	Oppenheimer, Beno W; Berger, Kenneth I; Segal, Leopoldo N; Stabile, A; Coles, K.	2014	https://www.proquest.com/docview/1494399689/2E1BA5ED485B4119PQ/2?accountid=48797 (accessed on 17 December 2021)
16	PROQUEST	Perioperative respiratory care in obese patients undergoing bariatric surgery: Implications for clinical practice [39]	Pouwels, S; Smeenk, F. W; Manschot, L; Lascaris, B; Nienhuijs, S; Bouwman, R. A; Buise, M. P.	2016	https://www.sciencedirect.com/science/article/pii/S0954611116301287 (accessed on 17 December 2021)
17	PROQUEST	Therapeutic strategies for the management of dry mouth with emphasis on electrostimulation as a treatment option [40]	Tulek, A; Mulic, A; Hogset, M; Utheim, T. P; Sehic, A.	2021	https://www.hindawi.com/journals/ijd/2021/6043488/ (accessed on 17 December 2021)
18	PROQUEST	Pulmonary vascular congestion: A mechanism for distal pulmonary unit dysfunction in obesity [41]	Oppenheimer, Beno W; Berger, Kenneth I; Saleem, Alí; Segal, Leopoldo N; Donnino, R.	2016	https://www.proquest.com/docview/1777735040/2E1BA5ED485B4119PQ/1?accountid=48797&forcedol=true (accessed on 17 December 2021)
19	PROQUEST	Comparison of Interview to Questionnaire for Assessment of Eating Disorders after Bariatric Surgery [4]	Globus, I; Kissileff, H. R; Hamm, J. D; Herzog, M; Mitchell, J. E; Latzer, Y.	2021	https://www.mdpi.com/2077-0383/10/6/1174 (accessed on 17 December 2021)
20	PROQUEST	Food insecurity as a determinant of obesity in humans: The insurance hypothesis [3]	Ortiga, D; Andrews, C; Bateson, M.	2017	https://www.proquest.com/docview/1988264531/144D2D2995EF403FPQ/471 (accessed on 17 December 2021)
21	PROQUEST	The impact of bariatric surgery on sleep-disordered breathing parameters from overnight polysomnography and home sleep apnea testing [13]	Mashaqi, S; Steffen, K; Crosby, R; Garcia, L; Cureus, P.	2018	https://www.proquest.com/docview/2080487482/8E81C0CD3DA94C5EPQ/198 (accessed on 17 December 2021)
22	PROQUEST	Perceived oral health in patients after bariatric surgery using measures of quality of life related to oral health [2]	Karlsson, L; Carlsson, J; Jenneborg, K; Kjaeldgaard, M.	2018	https://www.proquest.com/docview/2266411926/8E81C0CD3DA94C5EPQ/155 (accessed on 17 December 2021)
23	PROQUEST	Obstructive Sleep Apnea: A Focus on Myofunctional Therapy [42]	Felicio, C. M; Da Silva Dias, F; Voi Trawitzki, L.	2018	https://www.proquest.com/docview/2229610711/ECF01209F16B4AFCPQ/12 (accessed on 17 December 2021)
24	PROQUEST	Why is primary obesity a disease? [7]	De Lorenzo, A; Gratteri, S; Gualtieri, P; Cammarano, A; Bertucci, P.	2019	https://www.proquest.com/resultsol/144D2D2995EF403FPQ/28#scrollTo (accessed on 17 December 2021)
25	PROQUEST	Binge eating disorder and related features in candidates for bariatric surgery [43]	Cella, S., Fei, L; D’Amico, R; Giardiello, C; Allaria, A; Cotrufo, P.	2019	https://www.proquest.com/docview/2244691685/8E81C0CD3DA94C5EPQ/67 (accessed on 17 December 2021)
26	PROQUEST	Obesity, a risk factor in COVID-19 [5]	Cadea, E.	2021	https://www.minsalud.gov.co/Paginas/Obesidad-un-factor-de-riesgo-en-el-covid-19.aspx (accessed on 17 December 2021)
27	PROQUEST	Dysphagia symptoms in obstructive sleep apnea: Prevalence and clinical correlates [44]	Pizzorni, N; Radovanovic, D; Pecis, M; Lorusso, R; Annoni, F; Bartorelli, A; Santus, P.	2021	https://www.proquest.com/docview/2528901432/CDDFC8E76B784FF0PQ/20 (accessed on 17 December 2021)
28	PROQUEST	Obesity in the world [8]	Malo-Serrano, M; Castillo, N; Pajita, D.	2017	http://www.scielo.org.pe/scielo.php?pid=S1025-55832017000200011&script=sci_arttext (accessed on 17 December 2021)
29	PROQUEST	Social support for people with morbid obesity in a bariatric surgery program: A qualitative descriptive study [45]	Torrente-Sánchez et al.	2021	https://www.proquest.com/docview/2544977715/8E81C0CD3DA94C5EPQ/ (accessed on 17 December 2021)
30	PROQUEST	The development of eating and eating disorders after bariatric surgery: A systematic review and meta-analysis [14]	Victor Taba, J; Oliveira Suzuki, M; Sayuri do Nascimento, F; Ryuchi Iuamoto, L; Hsing, W. T; Zumerkorn Pipek, L; Andraus, W.	2021	https://www.proquest.com/docview/2554777851/8E81C0CD3DA94C5EPQ/28 (accessed on 17 December 2021)
31	PROQUEST	Design and Conduct of Systematic Reviews: A training guide for Early Literacy (EL) researchers [21]	Red Para La Lectoescritura Inicial De Centroamérica Y El Caribe–Redlei-	2021	https://red-lei.org/wp-content/uploads/2021/03/Directrices-de-Revisiones-Sistematicas.pdf (accessed on 17 December 2021)
32	PROQUEST	Imbalanced coagulation in the airways of Type 2-high asthma with comorbid obesity [46]	Womble, J. T; McQuade, V. L; Ihrie, M. D; Ingram, J. L.	2021	https://www.ncbi.nlm.nih.gov/pmc/articles/PMC8364356/ (accessed on 17 December 2021)
33	PUBMED	Obesity and the pulmonologist [47]	Deane, S; Thomson, A.	2006	https://adc.bmj.com/content/91/2/188.short (accessed on 13 January 2022)
34	PUBMED	Altered respiratory physiology in obesity [48]	Parameswaran, K; Todd, D. C; Soth, M.	2006	https://www.hindawi.com/journals/crj/2006/834786/ (accessed on 13 January 2022)
35	PUBMED	Obesity and respiratory diseases [49]	Zammit, C; Liddicoat, H; Moonsie, I; Makker, H.	2006	https://pubmed.ncbi.nlm.nih.gov/21116339/ (accessed on13 January 2022)
36	PUBMED	Altered respiratory physiology in obesity for the anesthesiologist-critical physician [50]	Porhomayon, J; Papadakos, P; Singh, A; Nader, N. D.	2011	https://pubmed.ncbi.nlm.nih.gov/23439281/ (accessed on 13 January 2022)
37	PUBMED	The stomatognathic system [51]	Mizraji, M; Freese, A. M; Bianchi, R.	2012	https://pesquisa.bvsalud.org/portal/resource/pt/lil-706324 (accessed on 13 January 2022)
38	PUBMED	Fundamental frequency, maximum phonation time, and vocal complaints in morbidly obese women [52]	Rocha de Souza, L. B; Medeiros Pereira, R; Marques dos Santos, M; Almeida Godoy, C. M.	2014	https://www.ncbi.nlm.nih.gov/pmc/articles/PMC4675479/ (accessed on 13 January 2022)
39	PUBMED	PICO tool for the formulation and search of clinically relevant questions in evidence-based psycho-oncology [24].	Landa-Ramírez, E; Arredondo, A.	2014	https://www.seom.org/seomcms/images/stories/recursos/05%20PSICOVOL11N2-3w.pdf (accessed on 13 January 2022)
40	PUBMED	Gastrointestinal symptoms in morbid obesity–the presence of dysphagia [53]	Huseini, M; Wood, G. C; Seiler, J; Argyropoulos, G; Irving, B. A; Gerhard, G. S; Rolston, D. D.	2014	https://pubmed.ncbi.nlm.nih.gov/25593922/ (accessed on 13 January 2022)
41	PUBMED	Speech, hearing, and language science therapy in bariatric surgery for the elderly: A case report [54]	Braude Canterji, M; Miranda Correa, S. P; Vargas, G. S; Ruttkay Pereira, J. L; Finard, S. A.	2015	https://www.scielo.br/j/abcd/a/zXYmf9QDgCCspDc5wyYdwHn/?lang=pt (accessed on 13 January 2022)
42	PUBMED	Respiratory management of obese patients undergoing surgery [55]	Hodgson, L. E; Murphy, P. B; Hart, N.	2015	https://www.ncbi.nlm.nih.gov/pmc/articles/PMC4454851/ (accessed on 14 January 2022)
43	PUBMED	Dysphagia after vertical sleeve gastrectomy: An evaluation of risk factors and evaluation of endoscopic intervention [56]	Nath, A; Yewale, S; Tran, T; Brebbia, J. S; Shope, T. R; Koch, T. R.	2016	https://pubmed.ncbi.nlm.nih.gov/28058017/ (accessed on 14 January 2022)
44	PUBMED	Monitoring of respiration and oxygen saturation in patients during the first night after elective bariatric surgery: A cohort study [57]	Wickerts, L; Forsberg, S; Bouvier, F; Jakobsson, J.	2017	https://pubmed.ncbi.nlm.nih.gov/28794858/ (accessed on 14 January 2022)
45	PUBMED	The pathogenesis of obesity: A scientific statement from an endocrine society [58]	Schwartz, M. W; Seeley, R. J; Zeltser, L. M; Drewnowski, A; Ravussin, E; Redman, L. M; Leibel, R. L.	2017	https://www.ncbi.nlm.nih.gov/pmc/articles/PMC5546881/ (accessed on 14 January 2022)
46	PUBMED	Positive airway pressure vs. inspiratory load exercises focused on pulmonary and respiratory muscle functions in the postoperative period of bariatric surgery [59]	Simões da Rocha, M. R; Souza, S; Moraes da Costa, C; Bertelli Merino, D. F; de Lima Montebelo, M. I; Rasera-Júnior; Pazzianotto-Forti, E. M.	2018	https://pubmed.ncbi.nlm.nih.gov/29972391/ (accessed on 13 January 2022)
47	PUBMED	Obesity, obstructive sleep apnea, and type 2 diabetes mellitus: Epidemiologic and pathophysiologic data [60]	Jehan, S; Myers, A. K; Zizi, F; Pandi-Perumal, S. R; Jean-Louis, G; McFarlane, S. I.	2018	https://www.ncbi.nlm.nih.gov/pmc/articles/PMC6112821/ (accessed on 15 January 2022)
48	PUBMED	Perioperative treatment of sleep-disordered breathing and outcomes in bariatric patients [61]	Meurgey, J. H; Brown, R; Woroszyl-Chrusciel, A; Steier, J.	2018	https://pubmed.ncbi.nlm.nih.gov/29445538/ (accessed on 13 January 2022)
49	PUBMED	Evaluation and management of obesity hypoventilation syndrome: An official clinical practice guideline of the American Thoracic Society [62]	Mokhlesi, B; Masa, J. F; Brozek, J. L; Gurubhagavatula, I; Murphy, P. B; Piper, A. J; Teodorescu, M.	2018	https://pubmed.ncbi.nlm.nih.gov/31368798/ (accessed on 13 January 2022)
50	PUBMED	Breathing matters [17]	Del Negro, C. A; Funk, G. D; Feldman, J. L.	2018	https://pubmed.ncbi.nlm.nih.gov/29740175/#affiliation-3 (accessed on 13 January 2022)
51	PUBMED	Duration and stability of metabolically healthy obesity over 30 years [6]	Camhi, S. M; Must, A; Gona, P. N; Hankinson, A; Odegaard, A; Reis, J; Carnethon, M. R.	2019	https://www.nature.com/articles/s41366-018-0197-8 (accessed on 13 January 2022)
52	PUBMED	Respiratory mechanics of patients with morbid obesity [63]	Sant’Anna, M. D; Ferreira Carvalhal, R; Bastos de Oliveira, F. D; Araújo Zin, W; Lopes, A. J; Lugon, J. R; Silva Guimarães, F.	2019	https://pubmed.ncbi.nlm.nih.gov/31644708/ (accessed on 13 January 2022)
53	PUBMED	Esophageal pathophysiologic changes and adenocarcinoma after bariatric surgery: A systematic review and meta-analysis [64]	Jaruvongvanich, V; Matar, R; Ravi, K; Murad, M. H; Vantanasiri, K; Wongjarupong, N; Dayyeh, B. K. A.	2020	https://www.ncbi.nlm.nih.gov/pmc/articles/PMC7447443/ (accessed on 13 January 2022)
54	PUBMED	Perception of dyspnea during inspiratory resistive load testing in obese subjects awaiting bariatric surgery [12]	Tomasini, K; Ziegler, B; Sanches, P. R. S; da Silva Junior, D. P; Thomé, P. R; Dalcin, P. D. T. R.	2020	https://pubmed.ncbi.nlm.nih.gov/32415112/ (accessed on 13 January 2022)
55	PUBMED	Orofacial functions and forces in healthy young and adult males and females [65]	Dantas Giglio, L; Felício, C. M. D; Voi Trawitzki, L. V.	2020	https://pubmed.ncbi.nlm.nih.gov/33174985/ (accessed on 13 January 2022)
56	PUBMED	Obesity, bariatric surgery, and periodontal disease: An update of the literature [66]	Franco, R; Barlattani Jr, A; Perrone, M. A; Basili, M; Miranda, M; Costacurta, M; Bollero, P.	2020	https://www.europeanreview.org/article/21196 (accessed on 13 January 2022)
57	PUBMED	Altered airway mechanics in the context of obesity and asthma [9]	Bates, J. H; Peters, U; Daphtary, N; MacLean, E. S; Hodgdon, K; Kaminsky, D. A; Dixon, A. E.	2021	https://pubmed.ncbi.nlm.nih.gov/33119471/ (accessed on 13 January 2022)
58	PUBMED	Pre-habilitation for bariatric surgery: A pilot study and randomized controlled trial protocol [67]	García-Delgado, Y; López-Madrazo-Hernández, M. J; Alvarado-Martel, D; Miranda-Calderín, G; Ugarte-Lopetegui, A; González-Medina, R. A; Wägner, A. M.	2021	https://pubmed.ncbi.nlm.nih.gov/34578781/ (accessed on 13 January 2022)
59	SCIELO	Bariatric surgery [16]	Maluenda, G. F.	2012	https://www.sciencedirect.com/science/article/pii/S0716864012702961 (accessed on 30 January 2022)
60	SCIELO	Myofunctional characteristics of obese mouth-nose breathers [68]	Bolzan Berlese, D; Ferreira Fontana, P. F; Botton, L; Maciel Weimnann, A. R; Bonfanti Haeffner, L. S.	2012	https://www.scielo.br/j/rsbf/a/NtJSY7LfJzPWyzRTsyN4cYL/?lang=pt (accessed on 30 January 2022)
61	SCIELO	Masticatory profile of morbidly obese subjects undergoing gastroplasty [69]	Marques Gonçalves, R. D. F; Zimberg Chehter, E.	2012	https://www.scielo.br/j/rcefac/a/cDMcdK4TtTt5WhQzpLR6pzD/?lang=pt (accessed on 30 January 2022)
62	SCIELO	The need for speech therapy assessment in the protocol of patients who are candidates for bariatric surgery [70]	Guerra Silva, A. S; Camargo Tanigute, C; Tessitore, A.	2014	https://www.scielo.br/j/rcefac/a/bHk9QNgbvFyXmw65QDJbzcD/?lang=pt (accessed on 30 January 2022)
63	SCIELO	Binge eating disorder [71]	Pinto de Azevedo, A. P; dos Santos, C. C; Cardoso da Fonseca, D.	2004	https://www.scielo.br/j/rpc/a/Mbjb77bcDLvBc4HPNgkT7Yn/?lang=pt (accessed on 30 January 2022)
64	SCIELO	Obesity, a risk factor in the COVID-19 pandemic [10]	de León Ramírez, L. L; de León Ramírez, L; Ramírez, J. A. B.	2021	http://www.revholcien.sld.cu/index.php/holcien/article/view/43 (accessed on 30 January 2022)
65	SCIELO	Chewing and swallowing in obese children and adolescents [19]	Souza, N. C. D; Ferreira Guedes, Z. C.	2016	https://www.scielo.br/j/rcefac/a/HypwyzNNyb9txHVPv5RX9 mJ/?lang=pt (accessed on 30 January 2022)
66	SCIELO	Speech therapy intervention in morbidly obese patients undergoing the Fobi-Capella gastroplasty method [72]	Marques Gonçalves, R. D; Zimberg, E.	2016	https://www.scielo.br/j/abcd/a/crM36KrQZBYR3W5d4RtrTfm/?lang=en (accessed on 30 January 2022)
67	SCIELO	Oropharyngeal dysphagia: Multidisciplinary solutions [73]	Álvarez Hernández, J.	2018	https://senpe.com/libros/01_DISFAGIA_INTERACTIVO.pdf (accessed on 30 January 2022)
68	SCIELO	Physiology of exercise in orofacial motricity: Knowledge of the subject [74]	Xavier Torres, G. M; Hernández Alvez, C.	2019	https://www.scielo.br/j/rcefac/a/dpdn39WnSLkbj5D3hhvhhqP/?lang=en (accessed on 30 January 2022)
69	SCIELO	Chewing and swallowing in obese individuals referred for bariatric surgery/gastroplasty: A pilot study [18]	Andrade Rocha, A. C; Oliveira De Souza, N; Davison Mangilli Toni, C.	2019	https://www.scielo.br/j/rcefac/a/5K4ZGD3QJr8PdhyLg6fpVQB/?lang=en (accessed on 30 January 2022)
70	SCIELO	Tooth wear and tooth loss in morbidly obese patients after bariatric surgery [75]	Duarte Aznar, F; Aznar, F. D; Lauris, J. R; Adami Chaim, E; Cazzo, E; Sales Peres, S. H. D. C.	2019	https://www.scielo.br/j/abcd/a/xdF3p8Fjb3vWrF9r9 mbDvVw/?lang=en (accessed on 30 January 2022)
71	SCIELO	Contributions of emotional overload, emotion dysregulation, and impulsivity to eating patterns in obese patients with binge eating disorder who seek bariatric surgery [15]	Benzerouk, F; Djerada, Z; Bertin, E; Barrière, S; Gierski, F; Kaladjian, A.	2020	https://www.mdpi.com/2072-6643/12/10/3099 (accessed on 30 January 2022)
72	SCIELO	Alimentary and bariatric surgery: Social representations of obese individuals [76]	Silva Gebara, T; Mocelin Polli, G; Wanderbroocke, A. C.	2021	https://www.scielo.br/j/pcp/a/6XkTBNs9MYqSPkkGnh3VJ5G/abstract/?format=html&lang=en (accessed on 02 February 2022)
73	SCIELO	Functional esophageal disorders in the preoperative evaluation of bariatric surgery [77]	Oliveira Lemme, E; Cerqueira Alvariz, A; Cotta Pereira, G.	2021	https://www.scielo.br/j/ag/a/Wh3kSvt3xqCQY7WRnZHFDjg/abstract/?lang=pt (accessed on 02 February 2022)
74	SCIELO	The relationship of sensory processing and the stomatognathic system in children who breathe through the mouth [78]	Dantas Lima, A. C. D; Costa Albuquerque, R; Andrade Cunha, D. A; Dantas Lima, C; Henrique Lima, S. J; Justino Silva, H.	2022	https://www.scielo.br/j/codas/a/yRRKqnrSx59xCdXFyT6hjCg/?lang=pt (accessed on 02 February 2022)
75	SPRINGER LINK	Obesity: Systemic and pulmonary complications, biochemical abnormalities, and impaired lung function [79]	Mafort, T. T; Rufino, R; Costa, C. H; Lopes, A. J.	2016	https://mrmjournal.biomedcentral.com/articles/10.1186/s40248-016-0066-z (accessed on 02 February 2022)
76	SPRINGER LINK	Obstructive sleep apnea and lung function in severely obese patients prior to and after bariatric surgery: A randomized clinical trial [80]	Aguiar, I. C; Freitas, W. R; Santos, I. R; Apostolico, N; Nacif, S. R; Urbano, J. J; Oliveira, L. V.	2014	https://mrmjournal.biomedcentral.com/articles/10.1186/2049-6958-9-43 (accessed on 02 February 2022)
77	SPRINGER LINK	Can myofunctional therapy increase tongue tone and reduce symptoms in children with sleep-disordered breathing? [81]	Villa, M. P; Evangelisti, M; Martella, S; Barreto, M; Del Pozzo, M.	2017	https://link.springer.com/article/10.1007/s11325-017-1489-2 (accessed on 02 February 2022)
78	SPRINGER LINK	Laryngopharyngeal reflux and dysphagia in patients with obstructive sleep apnea: Is there an association? [82]	Caparroz, F; Campanholo, M; Stefanini, R; Vidigal, T; Haddad, L; Bittencourt, L. R; Haddad, F.	2019	https://link.springer.com/article/10.1007/s11325-019-01844-0 (accessed on 02 February 2022)
79	SPRINGER LINK	Silent gastroesophageal reflux disease in morbidly obese patients prior to primary metabolic surgery [83]	Kristo, I; Paireder, M; Jomrich, G; Felsenreich, D. M; Fischer, M; Hennerbichler, F. P; Schoppmann, S. F.	2020	https://link.springer.com/article/10.1007/s11695-020-04959-6 (accessed on 02 February 2022)
80	SPRINGER LINK		Sandoval-Munoz, C. P; Haidar, Z. S	2021	https://link.springer.com/article/10.1186/s13005-021-00257-3 (accessed on 02 February 2022)

**Table 7:** Characterization of the stomatognathic system (morphological changes).

Obese Patient	Structure	Post-Bariatric Patient
Facial asymmetry with greater size in the middle and lower third of the face [18,30]	Face	Theoretical information not evidenced
Asymmetry in lip corners in normal position and in smile [65,70]		
Flattened and narrow nostrils (turbinate hypertrophy) [30,36,52]	Nose	Theoretical information not evidenced
Deviated septum [54]		
Tension with slight drop [18,70]	Cheeks	Theoretical information not evidenced
Hypertonia [18]		
Hypotonia [30,54]		
Dysfunction for inflating, retracting, and sucking [18]		
Atretic [30]	Maxillary	Theoretical information not evidenced
Impaired mobility [18,47]	Mandible	Theoretical information not evidenced
Clockwise rotation of the mandibular angle [30]		
Contraction during swallowing [18]	Lips	Theoretical information not evidenced
Dysfunction when protruding, retracting, and lateralizing on both sides [18]		
Decreased tone [30,54,69,70]		
Without lip seal [30,54]		
Short upper lip and functioning hiccups [30]		
Thick lower lip [15,29]		
Dryness [30]		
Mallampati scale with Class III (only the soft palate and uvula are visualized) and Class IV (only the hard palate is visualized) results [20] Dental caries [32] Open bite [30] Protrusion of upper teeth [66] Erosion, attrition, abrasion, and fraction [58] Loss of teeth [54]	Oral cavity	Invariance of Class III and IV on the Mallampati scale [20] Dental caries and dental erosion due to recurrent acidity in the oral cavity, with a higher prevalence in patients undergoing a Roux-en-Y gastric bypass [58]
	Teeth	Increased periodontal disease and hypersensitivity [2]
Abnormal position (lowered or low) [18,20,70]	Tongue	Theoretical information is not evidenced
Volume increase [18,20]		
Decreased tone (hypotonia) [30,54,69]		
Increased tone (hypertonia) [52,69]		
Difficulty performing praxis or movements [30,69]		
Tongue covered or interposed anteriorly between the dental arches [35,54]		
Dysfunction in the width and height of the hard palate (deep ogival) [18,70]	Palate	Theoretical information not evidenced
Soft palate with reduced mobility [30]		
Increased length of the soft palate [52]		
Presence of noise [18]	Temporomandibular articulation	Theoretical information not evidenced
Increase in circumference [42]	Neck	Theoretical information not evidenced
Presence of mental muscle tension during swallowing [18]	Muscles	Theoretical information not evidenced
Presence of reduced tone in temporalis muscle [70]		
Hypotonic orofacial musculature [30]		
Mental muscle hyperfunction [30]		
Excessive contraction of the orbicularis oris muscle [54]		
Left leaning posture in hyperextension [70]	Head	Theoretical information not evidenced
Hyperflexion [18]		
Right shoulder higher than left [70]	Shoulders	Theoretical information not evidenced
Narrowing due to accumulation of fat in the respiratory tract [20]	Larynx and pharynx	Theoretical information not evidenced
Thickening of the lateral walls with little possibility of seeing the posterior pharyngeal wall [11,20,52]		
Mechanical obstruction of the nasopharynx (adenotonsillar hypertrophy) [36]		
Pharyngeal collapse [81]		

**Table 8:** Characterization of the stomatognathic system (physiological changes).

Obese Patient	Function	Post-Bariatric Patient
Respiratory disorder with hypoventilation [13,57,62] Obstructive sleep apnea [13,18,20,40,57,61,62,69,81] Oral respiration [18,30,36] Reduced olfactory ability due to chronic nasal obstruction [68] Respiratory failure due to collapse of the upper airway [42,53] Lung disease and asthma [20] Decrease in the fundamental frequency due to obstruction of the air flow [52] Diaphragmatic dysfunction [59] Difficulty in phonorespiratory coordination [52] Presence of snoring [36] Presence of moderate dyspnea [12] Hypoxia [57] Oxygen desaturation [57] Reduced functional residual capacity [61]	Respiration	Significant reduction in obstructive sleep apnea-hypopnea index >50% and -20 events per hour [13] Mild respiratory disturbances [57] Presence of obstructive sleep apnea syndrome [57] Adult respiratory distress syndrome (ARDS) [57]
Escape during the emission of phonemes [70] Impaired spontaneous speech due to mandibular deviation [70] Short maximum phonation time [11] Altered voice quality (strangled, hoarse, and gasping) [11,79] Presence of hoarseness, murmurs, vocal instability, altered nervousness, and brilliance; in addition, strangulation of the voice at the end of the emission [11] Vocal fatigue with voice failure [37,52] Phonatory dysfunction due to dehydrated mucosa [40,56]	Phonation	No significant improvement in MPT or maximum phonation time [11]
Masticatory dysfunction due to: Dental alterations [2] Lack of bite force [18] Chronic and mild unilateral preference [18,19,44] Dehydrated mucosa [60] No presence of grinding phase [18] Hypotonicity of the lips and tongue [19] Rapid masticatory pattern [70,72] Taste reduction [80] Alternate bilateral chewing [30] Altered saliva production [40]	Mastication	Persistence of masticatory dysfunction
Swallowing dysfunction due to: Tension of facial muscles and altered posture [18] At the level of consistencies, 50% alteration in solids and 25% alteration in liquids [18] Dehydrated mucosa [40] Adapted swallowing [30] Low swallowing efficiency due to repeated swallowing of the bolus [18] Hypotonicity of the lips and tongue [20] Neural deterioration [83] Multiple swallows due to cheek hypotonicity [19] Difficulty in oral propulsion due to pharyngeal, nasal, or palatal obstruction [30] Presence of food residues in the cavity [19,70] Presence of large food bolus [70,72] Gastroesophageal reflux [44,81,83] Oropharyngeal dysphagia [84] Esophagitis [83] Swallowing dysfunction due to dehydrated mucosa [40] Binge eating disorder [43,71] Alteration in sense of taste and sensitivity of oral mucosa [77] Nutcracker esophageal dysphagia [81]	Swallowing	Sensation of choking or stagnation [18,70] Gastroesophageal reflux [2,18,70] after sleeve gastrectomy [83] Binge eating disorder [43,71] Acid reflux is increased in patients undergoing sleeve gastrectomy (SG) and decreased in patients undergoing Roux-en-Y gastric bypass (RYGB) [64]
Alteration of suction due to: Hypotonicity in cheeks [18] Impaired mouth breathing [30] Absence of lip seal with interposition of the tongue [54]	Suction	Theoretical information not evidenced

## Data Availability

Not applicable.
